# A Petal-type Chiral NADH Model: Design, Synthesis and its Asymmetric Reduction

**DOI:** 10.1038/srep17458

**Published:** 2015-12-09

**Authors:** Cui-Bing Bai, Nai-Xing Wang, Yan-Jing Wang, Yalan Xing, Wei Zhang, Xing-Wang Lan

**Affiliations:** 1 Technical Institute of Physics and Chemistry, Chinese Academy of Sciences, Beijing, 100190, China; 2 Department of Chemistry, William Paterson University of New Jersey, 300 Pompton Road, Wayne, New Jersey 07470, United States

## Abstract

A new type of NADH model compound has been synthesized by an efficient and convenient method. This model compound exhibits high reactivity and enantioselectivity in asymmetric reduction reactions. The results show that chiral NADH model **S** could be effectively combined with Mg^2+^ to form ternary complexes. This novel *C*_3_ symmetrical NADH model is capable of fluorescence emission at 460 nm when excited at 377 nm.

The study of Nicotinamide adenine dinucleotide (NADH) and its phosphate derivative (NADPH) is an active research field in organic chemistry and biochemistry^1^,^2^. It is of great significance for the research of redox reactions in biological system, such as photosynthesis, glycolysis, fatty acid synthesis, citric acid cycle and amino acid metabolism using the NADH molecules^3^. Since the pioneering work reported by Ohno and his co-workers in 1975^4^, many NADH models received particular attention and were widely explored in the past few decades^5^,^6^,^7^. Recently, Hantzsch esters have been widely and successfully used as reductant in the enantioselective transfer hydrogenation of unsaturated bonds with chiral catalyst, such as C=C and C=N, which can be successfully reduced in high yields under mild conditions^8^,^9^,^10^,^11^,^12^,^13^. However, compared to the various examples of catalytic asymmetric transfer hydrogenation reactions of C=C and C=N double bonds mediated by Hantzsch esters, fairly limited work has been reported on asymmetric hydrogenation reactions of C=O double bonds by chiral NADH models^13^. Therefore, design and synthesis of novel and efficient chiral NADH models for asymmetric reduction of C=O double bond with high enantioselectivity remain an urgent and challenging research topic.

To the best of our knowledge, the dihydropyridine amido group is the key structure in chiral NADH models and plays an important role in the hydride transfer process, so most of these biomimetic models carry an amide group at the 3-position of the 1,4-dihydropyridine^14^. Considerable efforts have been devoted to the preparation of diversely substituted chiral NADH models with the dihydropyridine amido group. From current voluminous literatures, we divide the chiral NADH models into three categories according to symmetry: 1) chiral NADH models with *C*_1_-symmetry^4^. Ohno and co-workers first disclosed that asymmetric reduction of prochiral alkylbenzoylformates by a chiral compound **1** and derivatives catalyzed by Mg^2+^ ion. 2) Chiral NADH models with *C*_2_-symmetry, such as compound **2** and **3** in Fig. 1^15^,^16^,^17^. Fig. 3) Chiral NADH models with *C*_3_-symmetry. In our previous work, we have designed and synthesized the first *C*_3_ symmetrical NADH model compound **4** with a special bowl-shaped conformation^18^.

In the past few decades, many chiral NADH models with *C*_1_ and *C*_2_-symmetry have been designed and synthesized respectively^19^,^20^,^21^,^22^,^23^. These models contain one or two dihydropyridine amido groups, which could not take full advantage of the dihydropyridine amido groups as chiral hydrogen sources. Meanwhile, many models are found unstable under natural conditions. Moreover, these model molecules usually have small size. The interaction with enzyme, such as dehydrogenase, could not achieve good matching and recognition. Therefore, to design novel NADH models with symmetric structure and excellent enantioselectivity is a challenge for chemists. For the *C*_3_-symmetry models, it was shown that the three identical dihydropyridine units were connected to form a rigidly defined concave cavity which could encase and fix certain substrates to accomplish the biomimetic reduction with high yields and enantioselectivity^18^.

Here in, as part of our ongoing studies with regards to the development of new chiral NADH models^18^,^24^,^25^,^26^, we would like to report a synthesis of a novel petal-type chiral NADH model, which has six chair carbon centers. This new *C*_3_ symmetrical NADH model shows better reactivity and enantioselectivity of asymmetric reduction of C=O bonds than the monomer portion with *C*_2_ symmetry (compound **2**). In addition, many experimental results show that in the presence of magnesium ion, chiral NADH model **S** could be combined with substrates to form ternary complexes. Also, the new NADH model has strong fluorescence emission phenomenon.

## Results and Discussion

**Figure 1:**
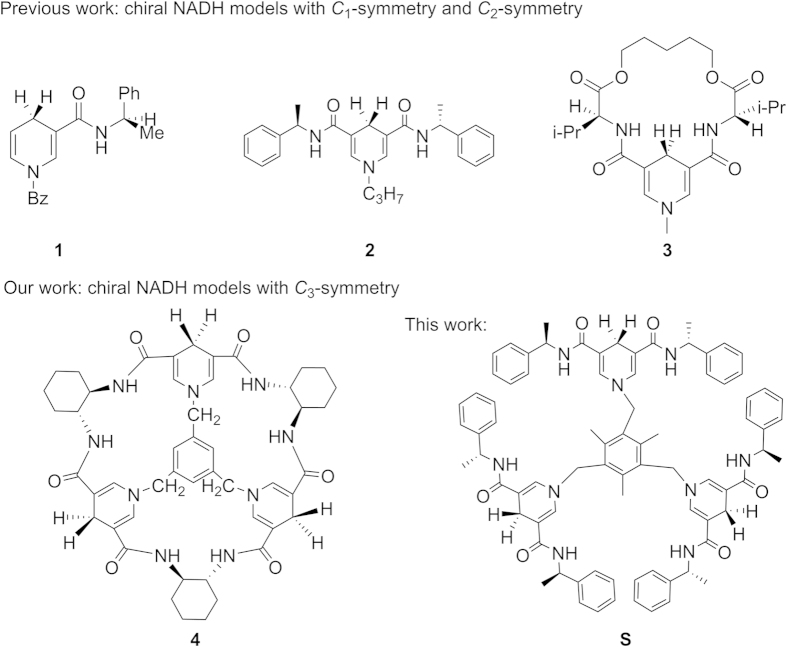
Representative chiral NADH model compounds with different *C*-symmetry.

**Figure 2:**
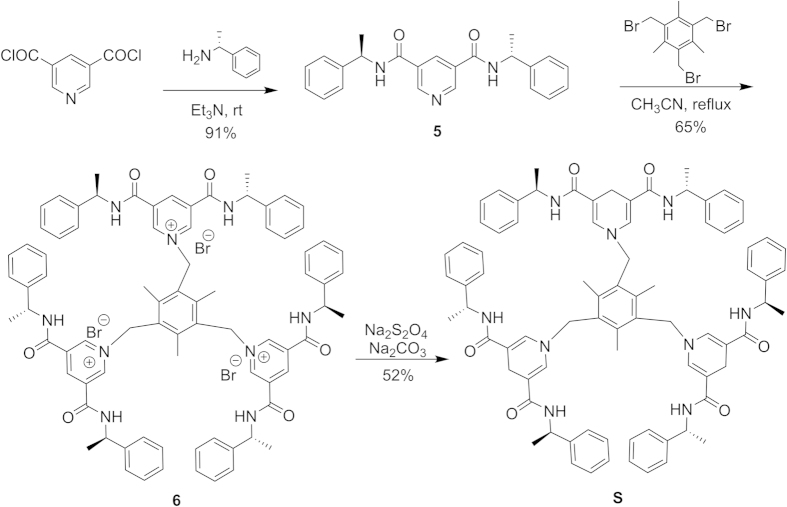
Synthesis of chiral NADH model **S**.

Inspired by Ohno’s introduction of (*R*)-α-methylbenzylamine into the NADH model **1** and the first *C*_3_ symmetrical NADH model **4** which are shown in Fig. 1, a new *C*_3_-symmetric chiral NADH model **S** bearing dihydropyridine amido group was synthesized. (*R*)-α-methylbenzylamine is introduced as the chiral source to connect three identical pyridine-3,5-dicarbonyl groups into three “petal”. Then three identical 1,4-dihydropyridine units are connected by the 1,3,5-tris(bromomethyl)-2,4,6-trimethylbenzene group to form model **S** (Fig. 2). Intermediate **5** was synthesized by the reaction of pyridine-3,5-dicarboxylic acid chloride with enantiomerically pure (*R*)-α-methylbenzylamine in a good yield. Treatment of **5** with 1,3,5-tris(bromomethyl)-2,4,6-trimethylbenzene generated pyridinium salts **6**^18^,^27^. Subsequently, regioselective reduction of the pyridinium salts **6** with sodium dithionite then gave the desired model **S**. To our delight, we found that model **S** is relatively stable, ^1^H NMR spectrum showed no signal changes after 6–12 hours when model **S** was kept under natural conditions (see Supplementary Fig. S1 online).

Our initial experiments were performed using methyl benzoylformate as a reduction substrate, and the effect of the model **S**/substrate/metal ratio was tested at room temperature. The results can be found as Supplementary Table S1 online. It was found that the reactivity and enantioselectivity of the asymmetric reduction reaction were the best when molar ratio of model **S**/substrate/metal was 1:1:1. The *ee* value was not affected significantly by the reaction time, but the yield increased with time. Using acetonitrile as solvent gave the desired products in good yields and high enantioselectivities. A more extensive study of the reduction reactions of model **S** showed that the enantioselectivity and the reactivity are dependent on metallic salts, especially magnesium salts. At room temperature, the yield of this reaction was low. Previous studies on the temperature effect of NADH model reactions showed that higher temperatures decreased the enantioselectivity^28^. Surprisingly, compound **7** was obtained with high *ee* (70%), although low yield by using CH_3_CN at −20 °C (Fig. 2). By comparison, this new *C*_3_ symmetrical NADH model shows better reactivity and enantioselectivity of asymmetric reduction of C=O bonds than the monomer portion compound **2** (less than 50% *ee*) in this reaction^15^. Encouraged by the good *ee*, we studied the reduction reaction of other related substrates at room temperature.

**Figure 3:**
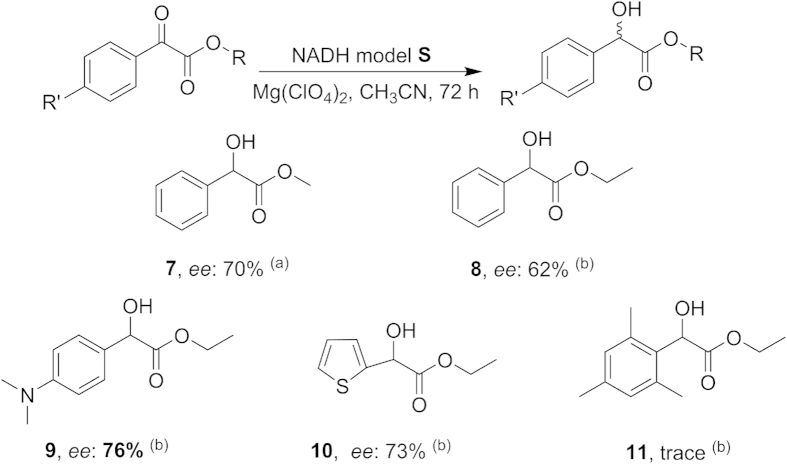
Asymmetric reduction reactions with model **S**. (**a**) The ratio of model / substrate / metal was1 : 1: 1, at -20 °C. (**b**) The ratio of model / substrate / metal was 1 : 1 : 1, at room temperature. Yield of isolated product. Enantiomeric excess was determined by chiral HPLC analysis.

To expand the scope of asymmetric reduction reaction of the petal-type chiral NADH model **S**, we studied the reduction reaction of ethyl benzoylformate (**8**), ethyl 4-dimethylaminobenzoylformate (**9**), ethyl thiophene-2-glyoxylate (**10**) and ethyl mesitylglyoxylate (**11**), as shown in Fig. 3. It is noteworthy that few people researched these compounds (**9**, **10** and **11**) in asymmetric reductions using chiral NADH models. To our delight, these compounds gave good enantioselectivities. We have previously studied substrates with electron-withdrawing group, such as ethyl 4-nitrophenylglyoxylate and ethyl 4-cyanobenzoylformate, however, only trace product was obtained and these compounds gave poor enantioselectivities.

Because the absence of complex formation between the *C*_2_ symmetrical NADH model **2** and magnesium ion, the monomer portion compound **2** was less efficient in the asymmetric reduction^15^. Thus, in order to better understand the complexation behavior between **S** with Mg^2+^, Mg^2+^ was added to the (CD_3_)_2_SO solution of **S**. We carefully investigated the job plot of compound **S** and Mg^2+^ using CIS (complexation induced shifts) of NMR measurement (see Supplementary Fig. S2 online). Upon complexation **S** with with Mg^2+^, proton peak (Hb) and the proton peak (Hc) are a little bit shifted to the low field with ^1^H NMR titration. In addition, the resulting mass spectra are shown in supporting information. A signal at m/z = 1307.6744 was observed in the positive mode that was assigned to [(**S**)Mg^II^+H]^+^ (calculated: 1307.6731) species (see Supplementary Fig. S3 online). Also, The chiral properties of model **S** were characterized by the CD spectra (see Supplementary Fig. S4 online) and model **S** show two broad CD features at long wavelength between 325 and 425 nm. All the results show that chiral NADH model **S** could be effectively combined with Mg^2+^ to form ternary complexes.

**Figure 4:**
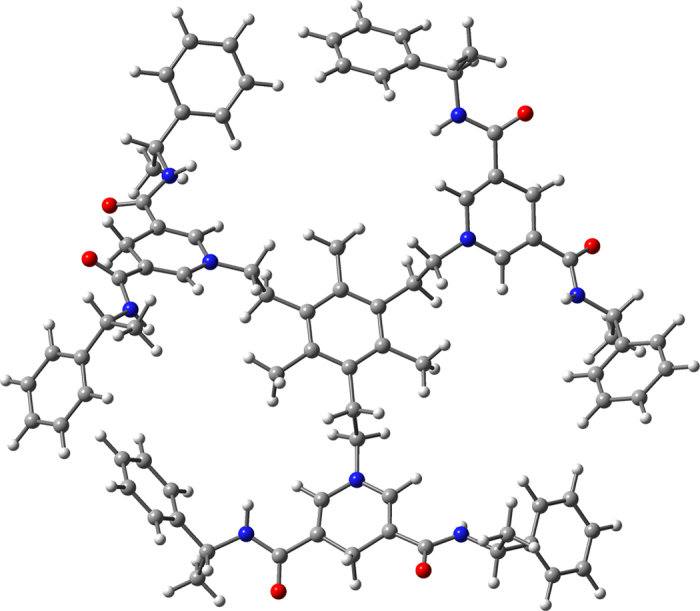
Chiral NADH model **S** conformation.

Furthermore, molecular modeling via molecular dynamics followed by energy minimization with Gaussian 03 demonstrated that the basin-shaped conformation shown in Fig. 4 is the most stable one. With 1,3,5-tris(bromomethyl)-2,4,6-trimethylbenzene ring as “pelvic floor”, three methyl groups stretch away in parallel. Three petal type structures composed by 1,4-dihydropyridine units, upward spiral around each other at the top of the benzene ring, formed a round and open “basin” structure. Besides, the concave cavity of the basin can hold a metal ion to from a transient and dynamic “ternary” complex in which the metal ion organizes the substrate and dihydronicotinamide for the hydride transfer^29^. Consistent with previously suggested transition-state models and theoretical calculations by energy minimization with Gaussian 03, we speculate that in the presence of magnesium ion, chiral NADH model **S** could be effectively combined with substrates to form ternary complexes.

It is well-known that NADH in nature is capable of fluorescent emission at 430–445 nm when excited at 340 nm, while the oxidized forms (NAD^+^) has not so, such as the oxidised form **6**^18^. The chiral NADH model **S** have not only a very good asymmetric reduction performance, but also excellent fluorescence features. We can use a chemical tool which exhibits both redox and fluorescence properties to solve a lot of chemistry problems. For example, for biochemical and supramolecular chemistry, a chemical tool which exhibits both redox and fluorescence properties can be used as fluorescence chemosensor for the detection of metal, which plays an important role in many biochemical processes at the cellular level. In addition, through the change in fluorescence intensity of NADH model **S**, we can detect the extent of the asymmetric reduction reaction. When the reaction is completed, the fluorescence intensity becomes weak. We believe that compounds which exhibit redox and fluorescence properties can be also applied to detect other redox reactions.

**Figure 5:**
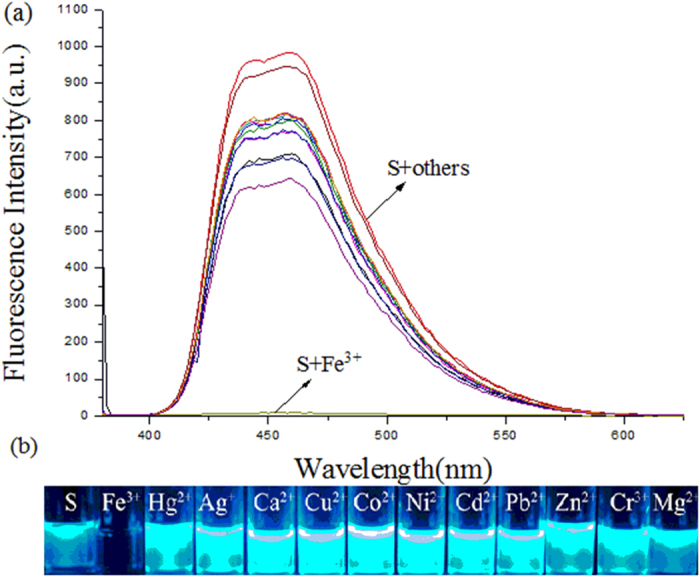
Fluorescence spectra changes of **S** in the presence of different metal ions.

In our previous paper, we have reported NADH model compound **4** displays fluorescent pH-sensing activity^18^. However, use of chiral NADH models for fluorescence chemosensor for the detection and measurement of metal ions are still very limited. To gain an insight into the fluorescent properties of receptor **S** toward various metal ions in DMSO solution, the emission changes were measured and the results were shown in Fig. 5. The emission of **S** appeared at the maximum emission wavelength was 460 nm in DMSO solution when excited at λ_ex_ = 377 nm (Fig. 5a). It is noteworthy that the maximum emission wavelength is higher than NADH model compound **4** and this is likely to be ever reported NADH models with the strongest fluorescence emission properties. When 20 equivalents of Fe^3+^ (4 × 10^−4^ M) was added to the DMSO solution of model **S**, dramatic fluorescent quenching was observed, the apparent fluorescence emission color change from bright blue to colorless was noticed by naked-eyes under UV irritation (Fig. 5b). In contrast, upon addition of other metal ions with their perchlorate salts, either no or slight decrease in intensity was observed. These results suggest that model **S** could be serve as a fluorescence chemosensor for Fe^3+^. More experiments of model **S** as fluorescent chemosensors are under exploration.

## Conclusion

In summary, we have designed and synthesized a novel petal-type NADH model with high reactivity and enantioselectivity in asymmetric reduction reactions. Asymmetric reduction of ethyl 4-dimethylaminobenzoylformate with model **S** produced ethyl 2-(4-(dimethylamino)phenyl)-2-hydr-oxyacetate in 76% *ee*, indicating that compound **S** was an efficient model of coenzyme NADH. As far as we know, this is the first report of NADH model as a fluorescent sensor for Fe^3+^ with good selectivity. The experimental results could provide a new strategy for the design of various NADH models based fluorescent chemosensors. Because of the flexible construction of the NADH model **S**, it may be combined with dehydrogenase well. Further work on applying dehydrogenase instead of magnesium ions to biological catalytic asymmetric reduction reaction with model **S** are also proceeding in our group.

## Methods

### General

All solvents and chemicals are used directly from commercial sources without further purification. Analytical Thin Layer Chromatography was carried out on precoated plates (silica gel 60), visualized with UV light. NMR spectra was performed on a Bruker DPX-400 spectrometer operating at 400 MHz (^1^H NMR) and 100 MHz (^13^C NMR). All spectra were recorded in CDCl_3_ or (CD_3_)_2_SO and the chemical shifts (δ) are reported in ppm relative to tetramethylsilane referenced to the residual solvent peaks. High-resolution mass spectral analyses (HRMS) were measured using ESI ionization. High-performance liquid chromatography (HPLC) analysis was performed on chiral column. All fluorescence spectra were recorded on a Shimadzu RF-5301 fluorescence spectrometer after the addition of perchlorate metal salts in DMSO, while keeping the ligand concentration constant (2.0 × 10^−5^ M). The excitation wavelength was 377 nm. Solutions of metal ions were prepared from the perchlorate salts of Fe^3+^, Hg^2+^, Ag^+^, Ca^2+^, Cu^2+^, Co^2+^, Ni^2+^, Cd^2+^, Pb^2+^, Zn^2+^, Cr^3+^ and Mg^2+^.

### General Procedure for the asymmetric reduction

The NADH model **S** (1 mmol), methyl benzoylformate (1 mmol) and magnesium perchlorate (1 mmol) were dissolved in acetonitrile (5 mL). The resulting solution was stirred in the dark under nitrogen at room temperature for 3 days. The reaction was quenched by adding 7–8 mL of water. The product was extracted with ethyl ether (3 × 10 mL) and the combined organic phases were dried over MgSO_4_, filtered and concentrated. The residue was purified by chromatography on silica gel (EtOAc: petrolum ether, 1:5 v/v) to give a white solid. Product identity and enantiomeric excess were determined by HPLC analysis using a Chiracel OD-H column. Chromatographic conditions: injection: 10 μL; eluent: n-hexane/2-propanol = 85:15; flow rate: 1.0 mL/min; UV detection: λ =  254 nm; Retention time: 5.737 min [(*S*)-enantiomer] and 8.212 min [(*R*)-enantiomer].

## Additional Information

**How to cite this article**: Bai, C.-B. *et al*. A Petal-type Chiral NADH Model: Design, Synthesis and its Asymmetric Reduction. *Sci. Rep*. **5**, 17458; doi: 10.1038/srep17458 (2015).

## Supplementary Material

Supplementary Information
