# Supplementary material for: A Petal-type Chiral NADH Model: Design, Synthesis and its Asymmetric Reduction
Source: Sci Rep. 2015 Dec 9;5:17458. doi: 10.1038/srep17458 (PMC4673579; doi:10.1038/srep17458)
Supplement: Supplementary Information [file srep17458-s1.doc]

A Petal-type Chiral NADH Model: Design, Synthesis and its Asymmetric Reduction

Cui-Bing Bai^1^, Yan-Jing Wang^1^, Yalan Xing^2*^, Wei Zhang^1^, Xing-Wang Lan^1^ & Nai-Xing Wang^1*^

^1^Technical Institute of Physics and Chemistry, Chinese Academy of Sciences, Beijing, 100190, China

^2^Department of Chemistry, William Paterson University of New Jersey, 300 Pompton Road, Wayne, New Jersey 07470, United States

Fax: +86-10-62554670; Tel: +86-10-82543575 [nxwang@mail.ipc.ac.cn](mailto:nxwang@mail.ipc.ac.cn)

**Table of Contents:**

1. Experimental section 1

2. Table S1. Explore the optimal conditions for reaction ..........................4

3. ^1^H and ^13^C NMR spectra and Figure S1.................................................5

4. HRMS spectra for **5**, **6** and model **S**.....................................................15

5. HPLC chart for **7** - **10**...........................................................................17

6. UV-vis spectra of model **S** in presence of Fe^3+^....................................20

7. Figure S2 - S4: ^1^H NMR titration, Mass spectra and CD spectra .......21

8. Data of molecular modeling.................................................................22

**1. Experimental section**

All solvents and chemicals are used directly from commercial sources without further purification. Analytical Thin Layer Chromatography was carried out on precoated plates (silica gel 60), visualized with UV light. NMR spectra was performed on a Bruker DPX-400 spectrometer operating at 400 MHz (^1^H NMR) and 100 MHz (^13^C NMR). All spectra were recorded in CDCl_3_ or CD_3_SOCD_3_ and the chemical shifts (δ) are reported in ppm relative to tetramethylsilane referenced to the residual solvent peaks. High-resolution mass spectral analyses (HRMS) were measured using ESI ionization. High-performance liquid chromatography (HPLC) analysis was performed on chiral column.

All fluorescence spectra were recorded on a Shimadzu RF–5301 fluorescence spectrometer after the addition of perchlorate metal salts in DMSO, while keeping the ligand concentration constant (2.0×10^-5^M). The excitation wavelength was 377 nm. Solutions of metal ions were prepared from the perchlorate salts of Fe^3+^, Hg^2+^, Ag^+^, Ca^2+^ , Cu^2+^, Co^2+^, Ni^2+^, Cd^2+^, Pb^2+^, Zn^2+^, Cr^3+^, and Mg^2+^.

***N^3^, N^5^-bis((R)-1-phenylethyl)pyridine-3,5-dicarboxamide, compound 5***

Pyridine-3,5-dicarboxylic acid (3.34 g, 20 mmol) was activated with an excess of SOCl_2_ (10 mL) at reflux. The reaction was carried out for 14 h, and then the excess of SOCl_2_ was removed under reduced pressure to obtain the acid chloride, which was used without further purification. A solution of acid chloride in 15 mL of CH_2_Cl_2_ was added to 25 mL of CH_2_Cl_2_ solution of (*R*)-1-phenylethylamine (5.45 g, 44.5 mmol) and Et_3_N (9 mL) with stirring at 0 ℃ (ice bath) for 2 h. The mixture was allowed to warm to room temperature and stirred for 12 h. The reaction mixture were washed with H_2_O, 1 N HCl, saturated aqueous NaHCO_3_, and brine. The organic phase was dried over MgSO_4_, filtered, and evaporated. The residue was washed with ethyl ether. Then the solid was filtered off and dried under vacuum overnight to give 6.81 g (91%) of compound **5** as a white solid. White solid, [α]_D_ = -23.0 (0.02 M in CH_3_OH), yield 91%; ^1^H NMR (400 MHz, (CD_3_)_2_SO) δ 9.18 (d, J = 10.88 Hz, 4H), 8.67 (s, 1H), 7.44-7.23 (m, 10H), 5.25-5.18 (m, 2H), 1.52 (d, J = 6.99 Hz, 6H); ^13^C NMR (100 MHz, (CD_3_)_2_SO): δ 164.17, 151.03, 144.93, 134.70, 130.21, 128.76, 127.20, 126.58, 49.22, 22.61; HRMS (ESI) m/z: [M+H]^+^ calcd for C_23_H_23_N_3_O_2_ 374.1863; found, 374.1861.

***1,1',1''-((2,4,6-trimethylbenzene-1,3,5-triyl)tris(methylene))tris(3,5-bis(((R)-1-phenylethyl)carbamoyl)pyridin-1-ium) bromide, compound 6***

In a first step, to a mixture of paraformaldehyde (16.7 g, 556.3 mmol) and trimethylbenzene (6.38g, 53.1 mmol) in 100 mL of HBr/AcOH (30 wt %) zinc bromide (19.7 g, 87.5 mmol) was slowly added at room temperature. The mixture was heated to 90 ℃ for 16.5 h, during which time white crystals were formed. The reaction was cooled to room temperature, and the white solid was filtered off, washed with water, and dried under vacuum overnight to give 1,3,5-tris(bromomethyl)-2,4,6-trimethylbenzene (20.06 g, 50.4 mmol, 95%) as a white solid. In a second step, A solution of compound **5** (1.0 g, 2.67 mmol) and 1,3,5-tris(bromomethyl)-2,4,6-trimethylbenzene (0.338 g, 0.85 mmol) in acetonitrile(15 mL) was stirred under reflux for 12 h and then cooled to room temperature. After evaporation of acetonitrile under reduced pressure, the residue was subjected to flash chromatography (CH_2_Cl_2_ / CH_3_OH 20:1) to give compound **6** as a yellow solid (0.84 g, 65%). Yellow solid, [α]_D_ = -20.9 (0.016 M in CH_3_OH), yield 65%; ^1^H NMR (400 MHz, (CD_3_)_2_SO): δ 9.71 (d, *J* = 7.77 Hz, 9H), 9.22 (s, 6H), 7.46 (d, *J* = 7.41 Hz, 12H), 7.35 (t, *J* = 7.53 Hz, 12H), 7.26 (t, *J* = 7.27 Hz, 6H), 6.20 (s, 6H), 5.21-5.14 (m, 6H), 2.42 (s, 9H), 1.56 (d, *J* = 7.01 Hz, 18H). ^13^C NMR (100 MHz, (CD_3_)_2_SO): δ 160.71, 145.70, 145.46, 144.41, 142.26, 134.30, 129.07, 128.99, 127.64, 127.01, 60.11, 50.40, 22.69, 18.25; HRMS (ESI) m/z: [M]^3+^ calcd for C_27_H_28_N_3_O_2_Br 426.2176; found, 426.2171.

***1,1',1''-((2,4,6-trimethylbenzene-1,3,5-triyl)tris(methylene))tris(N^3^,N^5^-bis((R)-1-phenylethyl)-1,4-dihydropyridine-3,5-dicarboxamide), chiral NADH model S***

A 25mL round-bottomed flask containing the pyridinium salt **6** (0.30 g, 0.197 mmol) and acetonitrile (10 mL) was placed under nitrogen in the dark. A solution of sodium dithionite (2.06 g, 11.83 mmol) and sodium carbonate (0.80 g, 7.55 mmol) in degassed water (10 mL) was added dropwise through a syringe. The reaction mixture was stirred for 24h at room temperature. After addition of water, the aqueous layer was extracted with CH_2_Cl_2_. The combined organic layers were dried over MgSO_4_. After separation on Sephadex LH-20 with methanol as the eluent, the pure chiral NADH model S was obtained. Yellow solid, yield 52%; ^1^H NMR (400 MHz, (CD_3_)_2_SO): δ 7.50 (d, *J* = 7.73 Hz, 6H), 7.33-7.29 (m, 24H), 7.23-7.20 (m, 6H), 6.88 (s, 6H), 5.09-5.02 (m, 6H), 4.58 (s, 6H), 3.28 (s, 6H), 2.36 (s, 9H), 1.40 (d, *J* = 6.98 Hz, 18H). ^13^C NMR (100 MHz, (CD_3_)_2_SO): δ 166.33, 145.72, 139.75, 135.34, 131.68, 128.76, 127.05, 126.71, 106.46, 55.49, 51.84, 48.50, 22.76, 17.32; HRMS (ESI) m/z: [M+H]^+^ calcd for C_81_H_87_N_9_O_6_ 1282.6852; found 1282.6857.

***General Procedure for the asymmetric reduction:*** The NADH model **S** (1 mmol), methyl benzoylformate (1 mmol) and magnesium perchlorate (1 mmol) were dissolved in acetonitrile (5 mL). The resulting solution was stirred in the dark under nitrogen at room temperature for 3 days. The reaction was quenched by adding 7-8 mL of water. The product was extracted with ethyl ether (3×10 mL) and the combined organic phases were dried over MgSO_4_, filtered and concentrated. The residue was purified by chromatography on silica gel (EtOAc / petrolum ether 1:5) to give a white solid. Product identity and enantiomeric excess were determined by HPLC analysis using a Chiracel OD-H column. Chromatographic conditions: injection: 10 µL; eluent: n-hexane/2-propanol = 85:15; flow rate: 1.0 mL/min; UV detection: λ= 254 nm; Retention time: 5.737 min [(*S*)-enantiomer] and 8.212 min [(*R*)-enantiomer].

***Characterization data:***

Compound **7**^[1]^: ^1^H NMR (400 MHz, CDCl_3_): δ 7.43-7.32 (m, 5H), 5.18 (s, 1H), 3.75 (s, 3H); ^13^C NMR (100 MHz, CDCl_3_): δ 174.14, 138.25, 128.64, 128.53, 126.61, 72.91, 53.05. The enantiomeric excess (*ee*) was determined by HPLC on a Chiralcel OD-H column (*n*-hexane / isopropanol = 85 / 15, flow rate 1 mL / min, λ= 254 nm), t_1_ = 5.737 min, t_2_ = 8.212 min.

Compound **8**^[2]^:^1^H NMR (400 MHz, CDCl_3_) δ 7.44-7.28 (m, 5H), 5.19 (s, 1H), 4.24-4.14 (m, 3H), 1.18 (t, *J* = 7.14 Hz, 3H); ^13^C NMR (100 MHz, CDCl_3_): δ 173.82, 138.53, 128.52, 128.35, 126.64, 73.02, 62.14, 13.98. The enantiomeric excess (*ee*) was determined by HPLC on a Chiralcel OD-H column (*n*-hexane / isopropanol = 90 / 10, flow rate 0.5 mL / min, λ= 254 nm), t_1_ = 12.480 min, t_2_ = 21.808 min.

Compound **9**^[3]^:^1^H NMR (400 MHz, CDCl_3_) δ 7.26 (d, *J* = 1.87 Hz, 2H), 6.71 (d, *J* = 8.78 Hz, 2H), 5.06 (d, *J* = 5.67 Hz, 1H), 4.27-4.16 (m, 2H), 3.25 (d, *J* = 5.95 Hz, 1H), 2.95 (s, 6H), 1.23 (t, *J* = 7.14 Hz, 3H); ^13^C NMR (100 MHz, CDCl_3_): δ 174.19, 150.65, 127.56, 126.20, 112.37, 72.75 61.92, 40.48, 14.10. The enantiomeric excess (*ee*) was determined by HPLC on a Chiralcel OD-H column (*n*-hexane / isopropanol = 95 / 5, flow rate 1 mL / min, λ= 254 nm), t_1_ = 14.924 min, t_2_ = 24.859 min.

Compound **10**^[2]^: ^1^H NMR (400 MHz, CDCl_3_) δ 7.27 (d, *J* = 5.07 Hz, 1H), 7.09 (d, *J* = 3.44 Hz, 1H), 6.98 (dd, *J* = 4.84, 3.78 Hz, 1H), 5.40 (d, *J* = 6.43 Hz, 1H), 4.3-4.2 (m, 2H), 3.66 (d, *J* = 5.70 Hz, 1H), 1.28 (t, *J* = 7.15 Hz, 3H); ^13^C NMR (100 MHz, CDCl_3_): δ 172.58, 141.75, 127.03, 125.76, 125.44, 69.30, 62.60, 14.15. The enantiomeric excess (*ee*) was determined by HPLC on a Chiralcel OD-H column (*n*-hexane / isopropanol = 90 / 10, flow rate 0.5 mL / min, λ= 254 nm), t_1_ = 15.037min, t_2_ = 21.384 min.

[1] N.-X. Wang, J. Zhao, *Adv. Synth. Catal.* **2009**, *351*, 3045-3050.

[2] Q.-H Meng, Y.-H Sun, R.-V. Virginie, J. P. Genêt, Z.-G. Zhang, *J. Org. Chem.* **2008**, *73*, 3842-3847.

[3] K. Aikawa, Y. Hioki, K. Mikami, *Chem. Asian J.* **2010**, *5*, 2346-2350.

1. **Table S1. Explore the optimal conditions for reaction.**

| Entry | Salts | **S** / Sub./ Salts | Solvent | T  (°C) | t  (h) | Yield (%)^[a]^ | % *ee*. (Conf.)^[b]^ |
| --- | --- | --- | --- | --- | --- | --- | --- |
| 1 | Mg(ClO_4_)_2_ | 1:3:3 | CH_3_CN | rt | 72 | 28 | 34 (S) |
| 2 | Mg(ClO_4_)_2_ | 1:3:1.5 | CH_3_CN | rt | 72 | 31 | 39 (S) |
| 3 | Mg(ClO_4_)_2_ | 1:3:1 | CH_3_CN | rt | 72 | 36 | 50 (S) |
| 4 | Mg(ClO_4_)_2_ | 1:2:1 | CH_3_CN | rt | 72 | 40 | 58 (S) |
| 5 | Mg(ClO_4_)_2_ | 1:1:1 | CH_3_CN | rt | 72 | 46 | 60 (S) |
| 6 | Mg(ClO_4_)_2_ | 1:1:1 | CH_3_CN | rt | 24 | 32 | 55 (S) |
| 7 | Mg(ClO_4_)_2_ | 1:1:1 | CH_3_CN | rt | 48 | 39 | 58 (S) |
| 8 | Mg(ClO_4_)_2_ | 1:1:1 | CH_3_CN | 0 | 72 | 26 | 62 (S) |
| **9** | **Mg(ClO_4_)_2_** | **1:1:1** | **CH_3_CN** | **-20** | **72** | **10** | **70 (S)** |
| 10 | Mg(ClO_4_)_2_ | 1:1:1 | CH_3_CN | 40 | 72 | 55 | 53 (S) |
| 11 | Mg(ClO_4_)_2_ | 1:1:1 | CH_2_Cl_2_ | rt | 72 | 17 | 32 (S) |
| 12 | Mg(ClO_4_)_2_ | 1:1:1 | Et_2_O | rt | 72 | 25 | 50 (S) |
| 13 | Mg(ClO_4_)_2_ | 1:1:1 | PhCH_3_ | rt | 72 | 23 | 30 (S) |
| 14 | Zn(ClO_4_)_2_  ·6H_2_O | 1:1:1 | CH_3_CN | rt | 72 | 11 | 14 (S) |
| 15 | Cu(OTf)_2_ | 1:1:1 | CH_3_CN | rt | 72 | 21 | 43 (S) |
| 16^[c]^ | Mg(ClO_4_)_2_ | 1:1:1 | CH_3_CN | rt | 72 | 23 | 25 (S) |

[a] Yield of isolated product. [b] Enantiomeric excess was determined by chiral HPLC analysis.[c] Compound **2** (*C*_2_ symmetry) was uesd in the reaction.

**3. ^1^H and ^13^C NMR spectra**

**compound 5:**


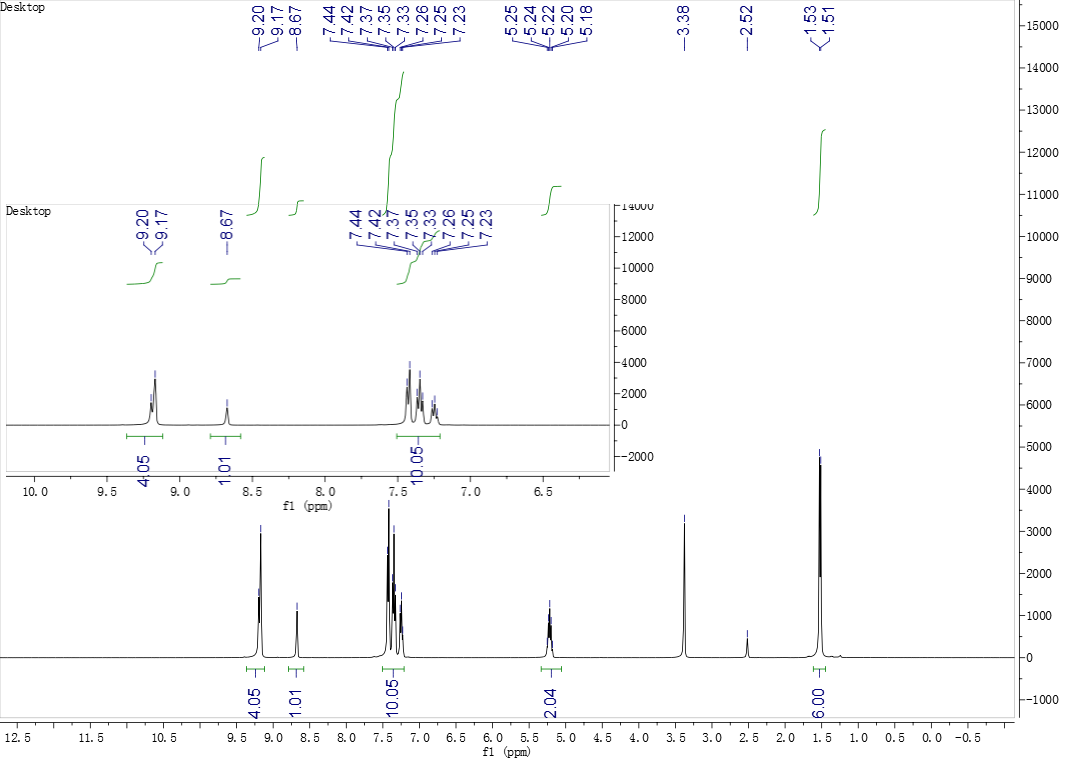


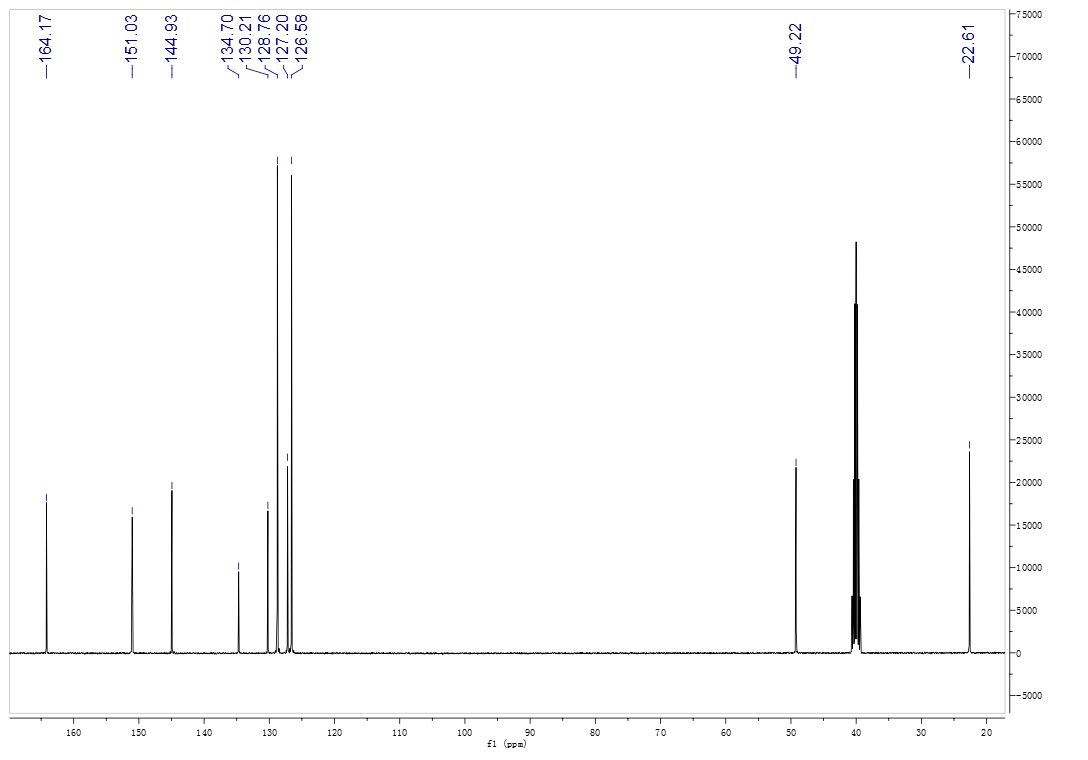


**compound 6:**


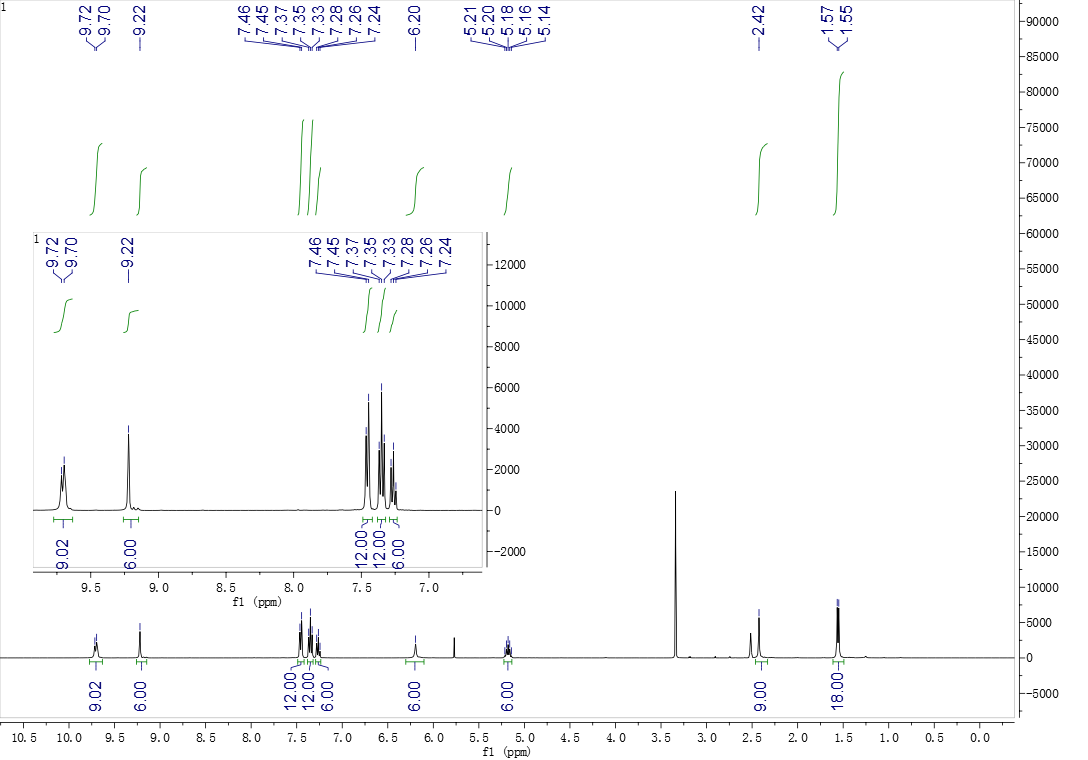


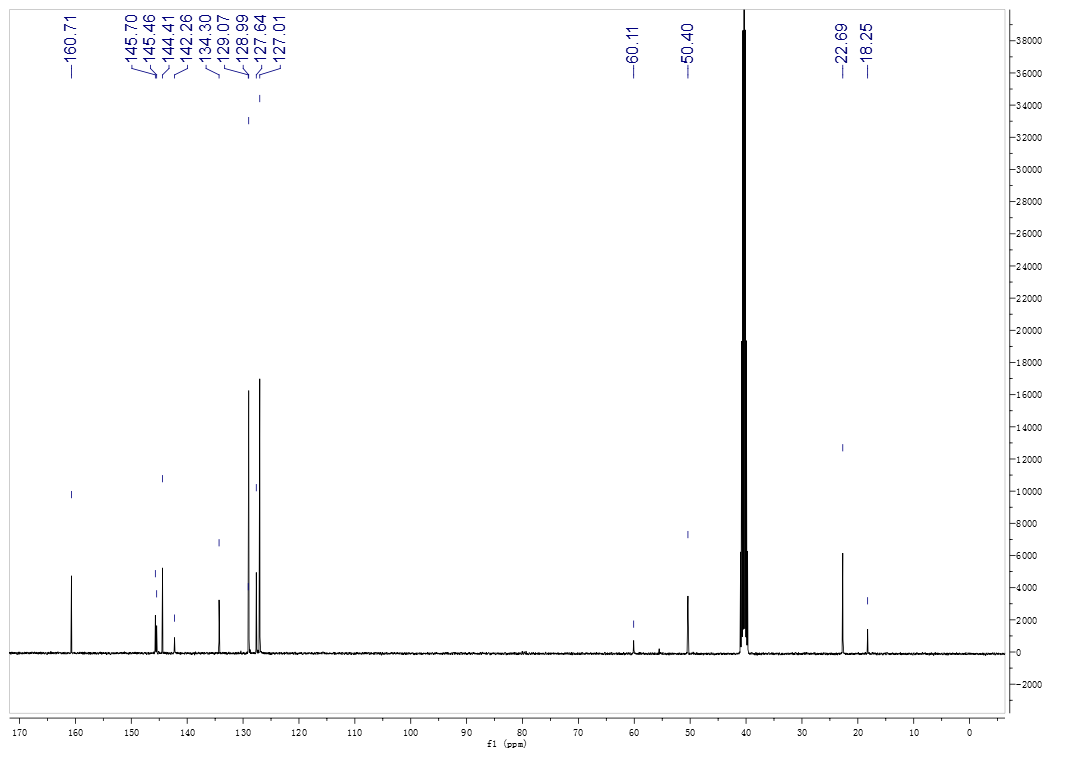


**chiral NADH model S**:


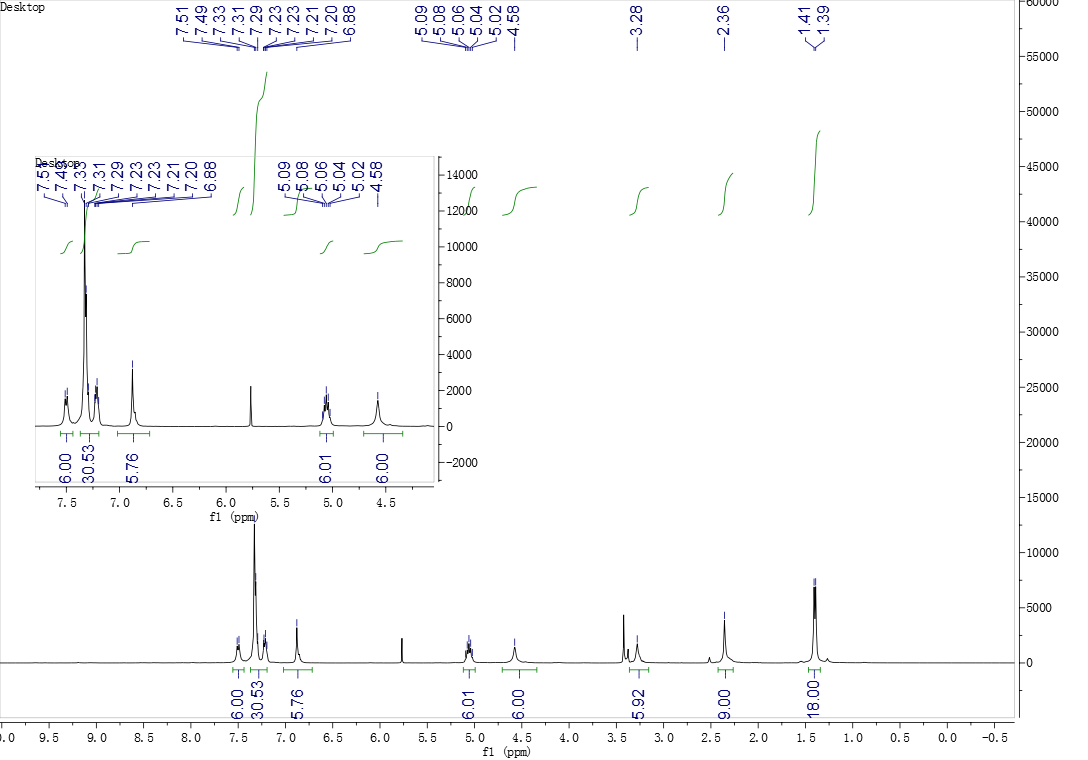


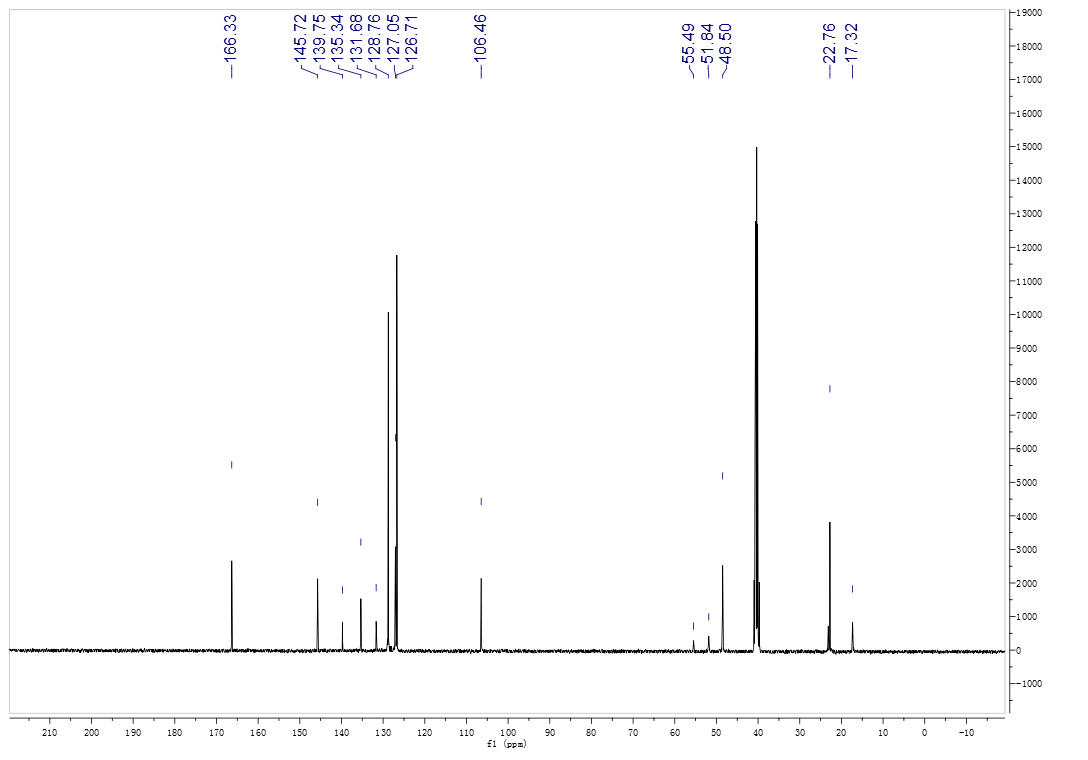


**compound 7:**


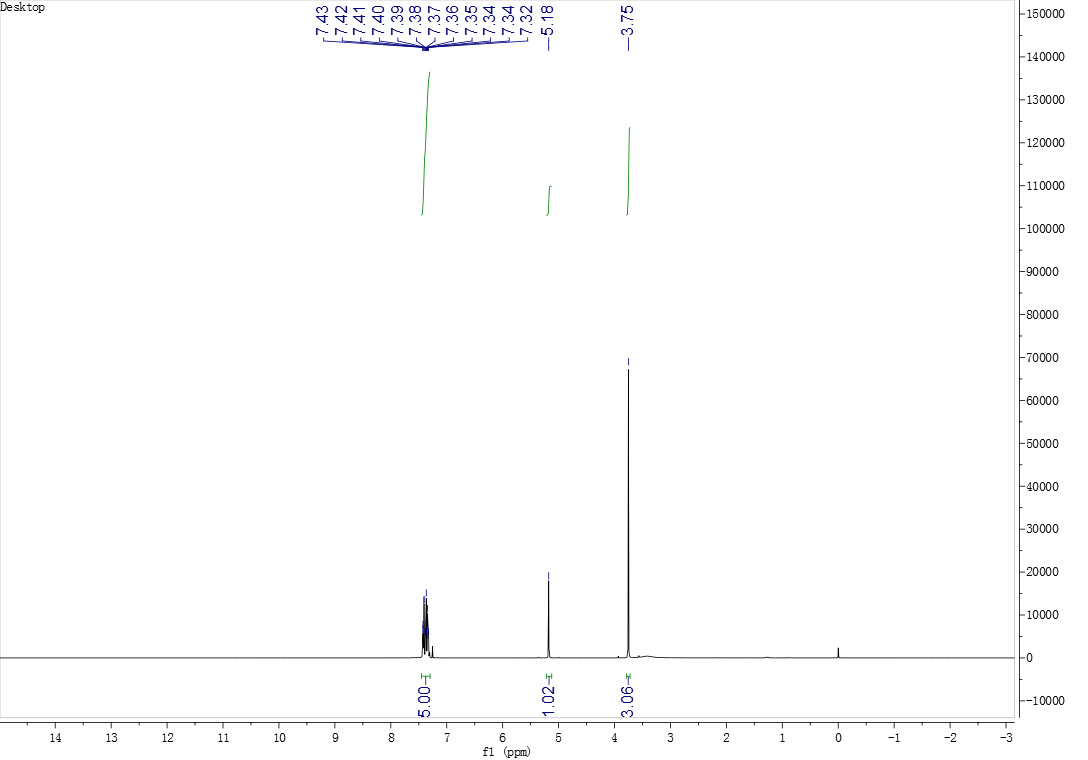


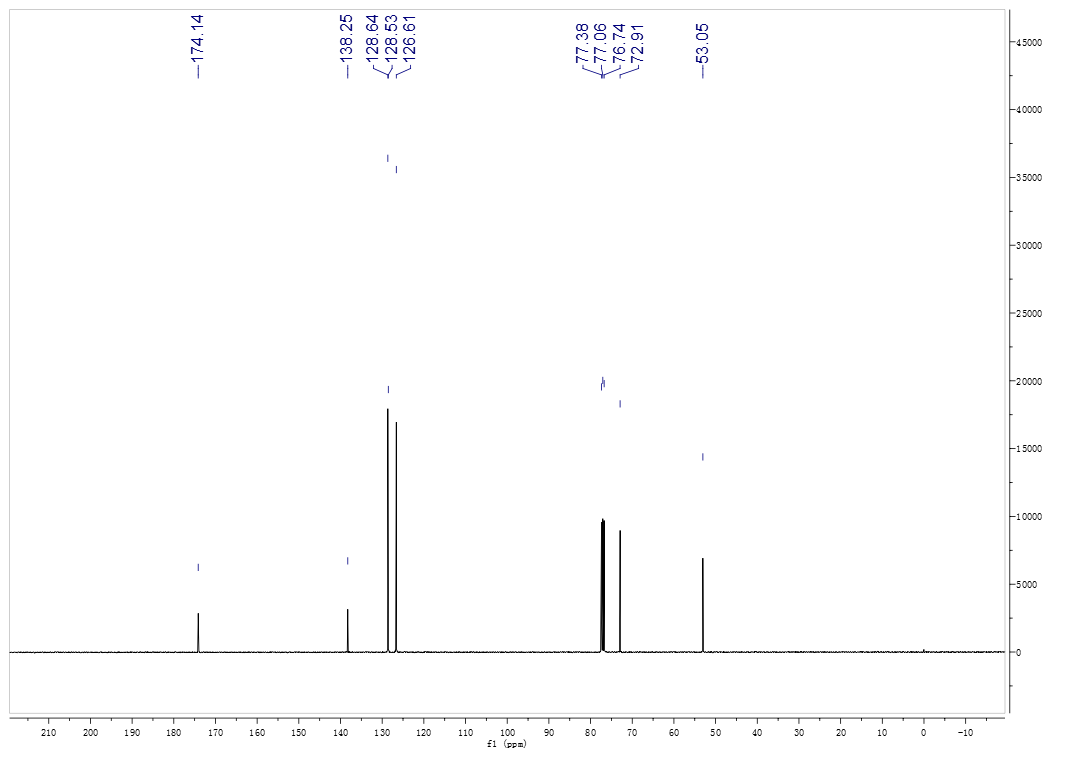


**compound 8:**


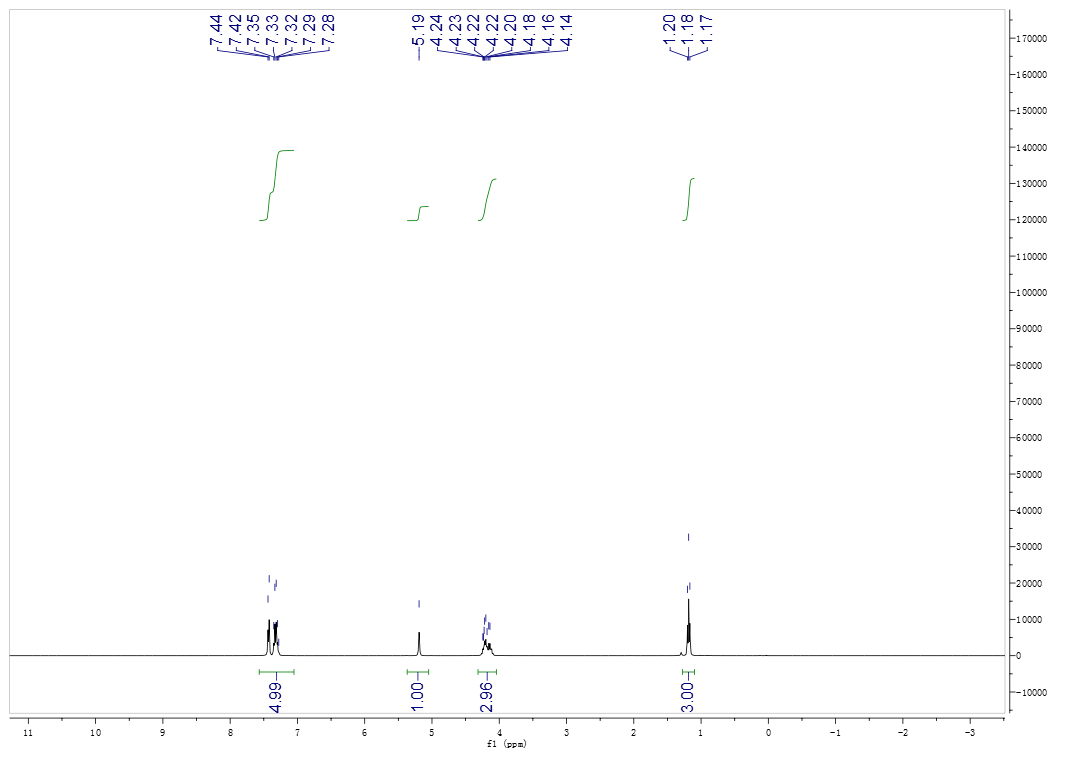


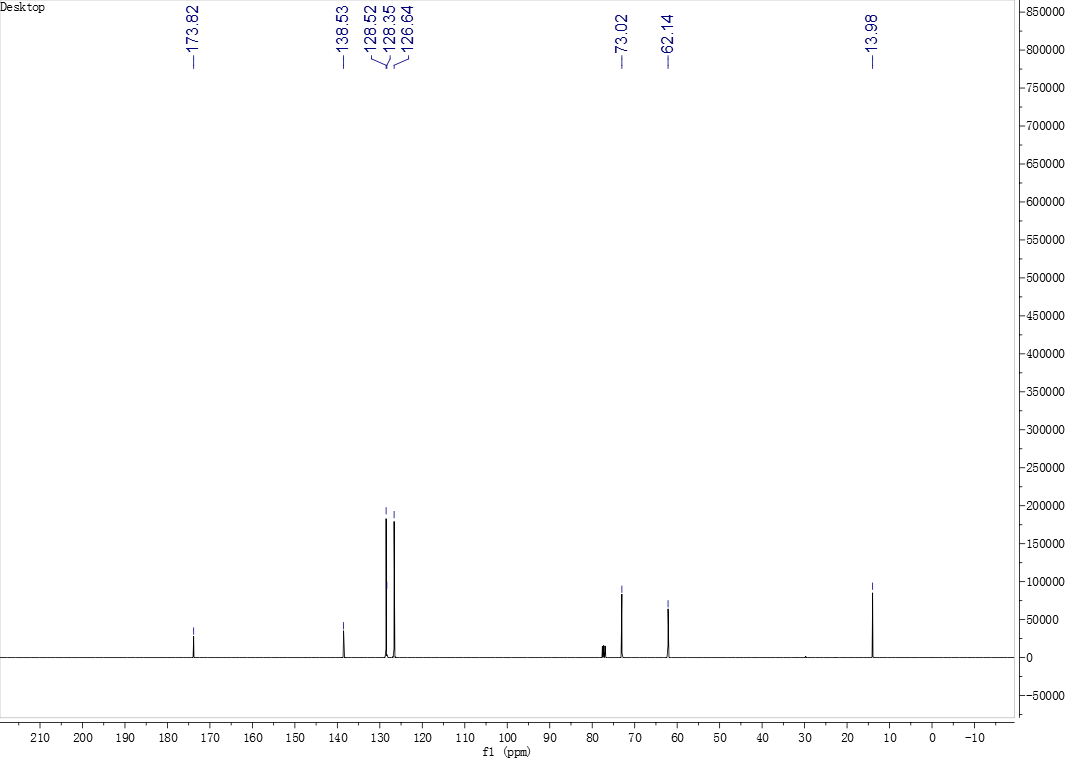


**compound 9:**


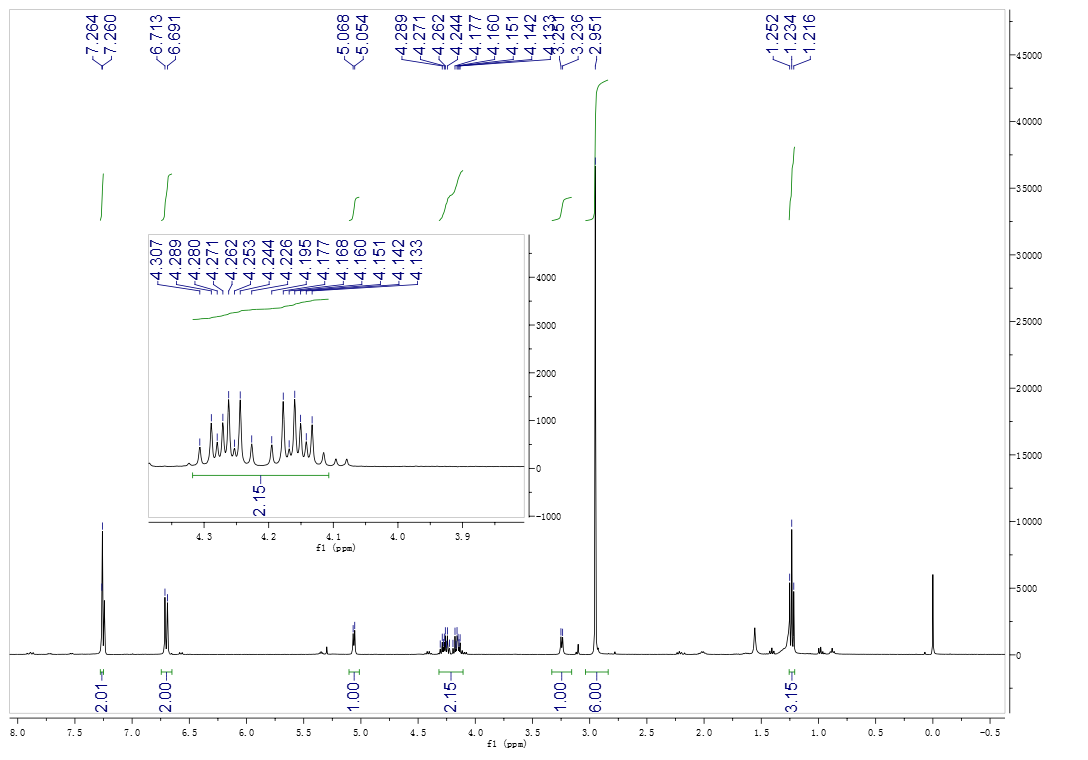


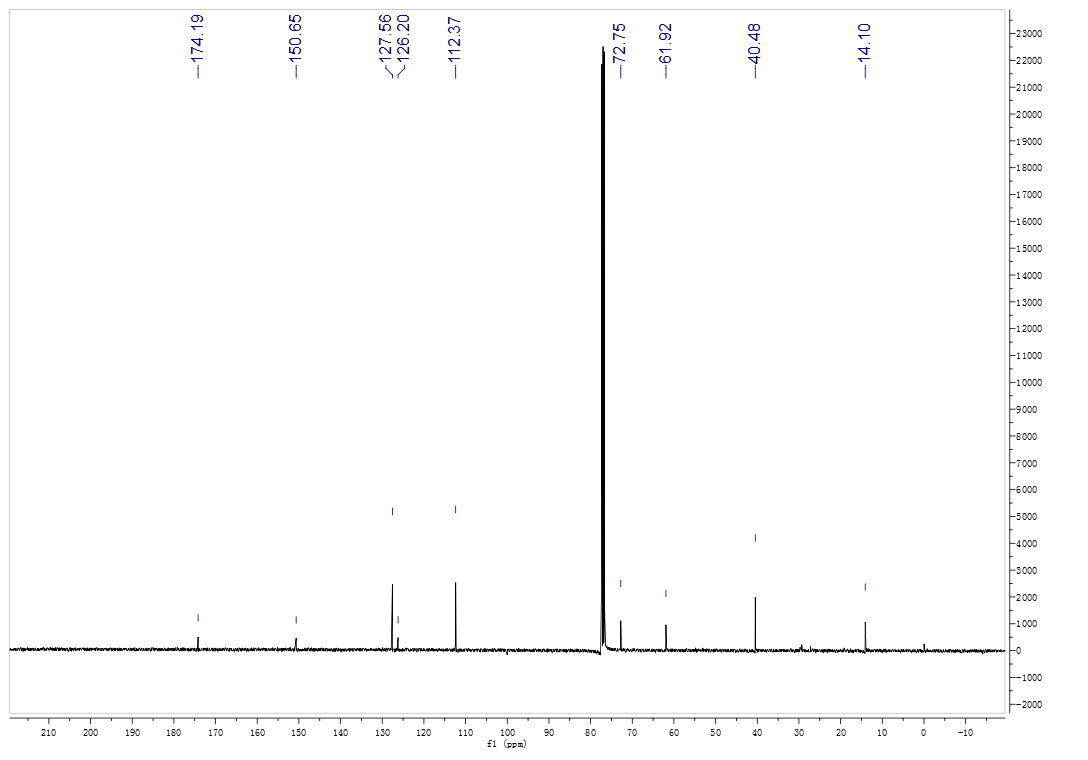


**compound 10:**


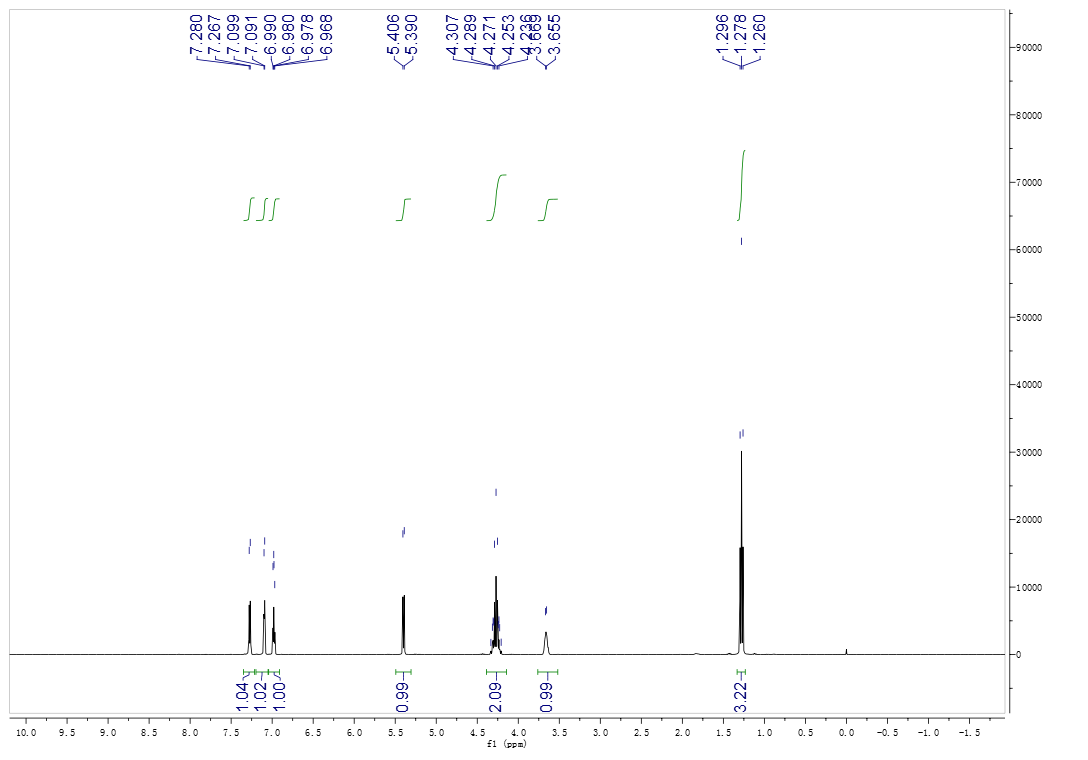


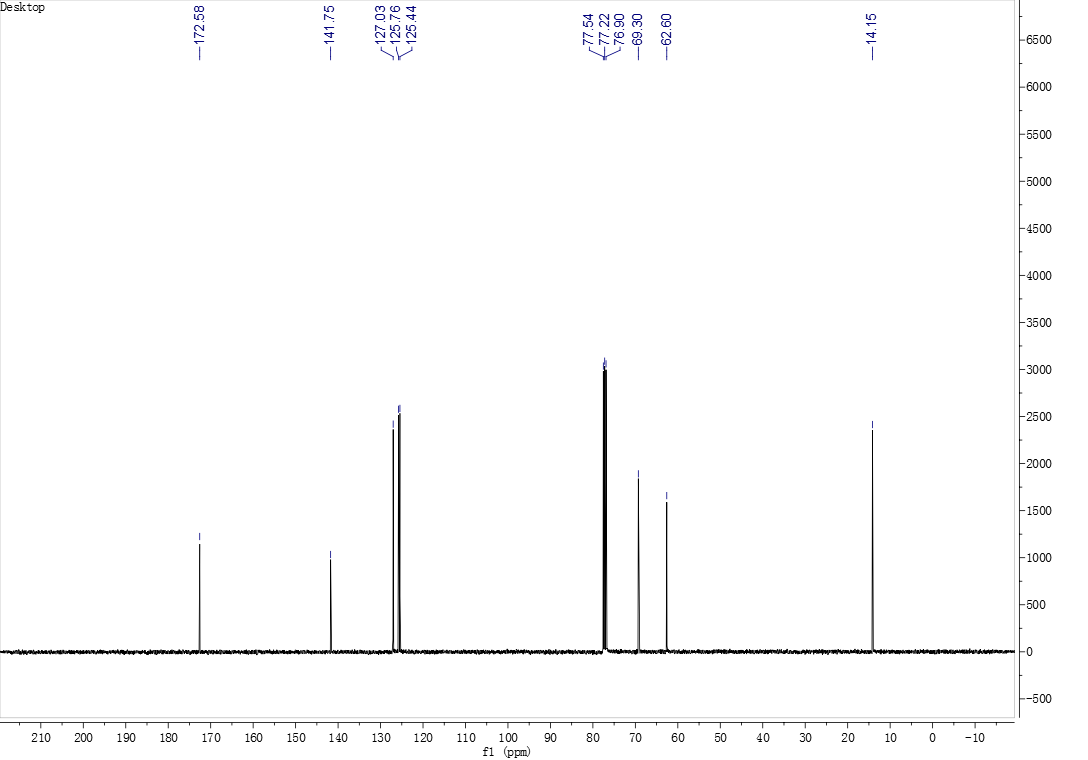


**Figure S1. ^1^H NMR Stability of Chiral NADH model S.**


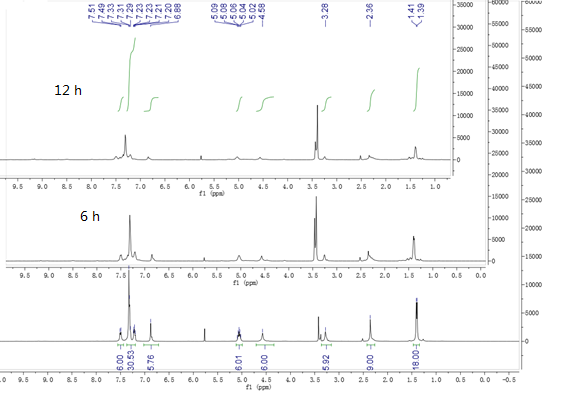


**4. HRMS spectra**

**compound 5:**

**compound 6:**

**chiral NADH model S :**

**5. HPLC chart for compound 7 - 10**

Racemic of **7**


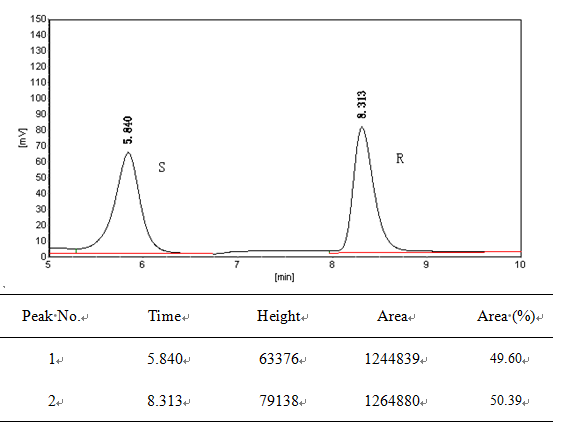


Table 1, entry 5：


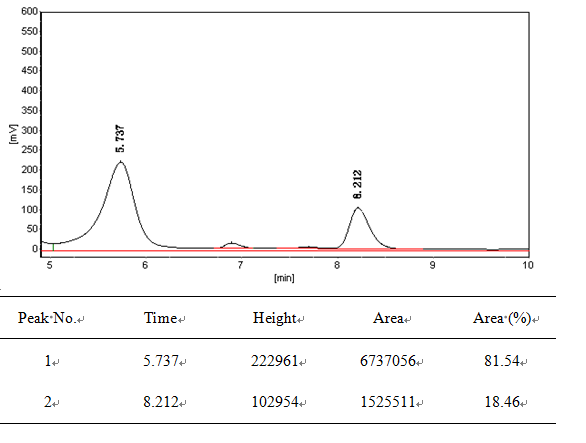


Table 1, entry 9：


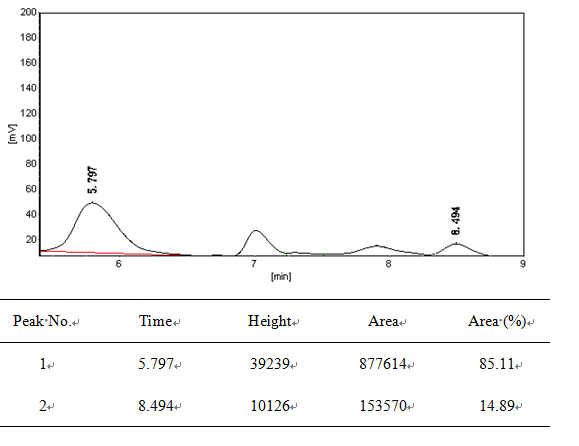


Racemic of **8**


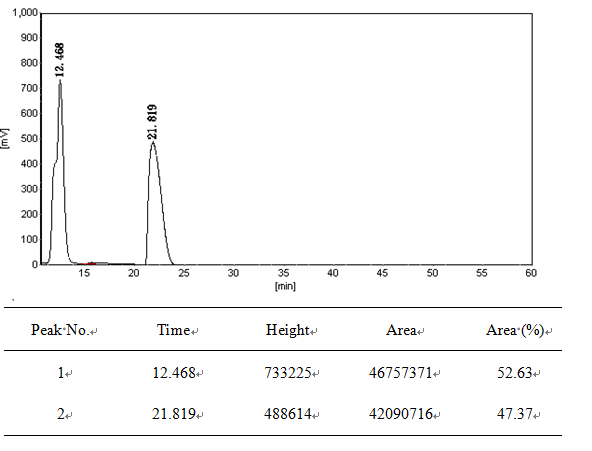


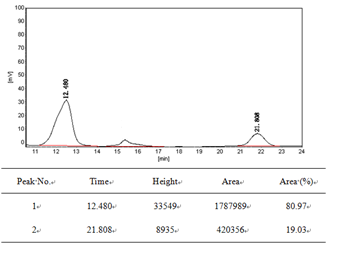


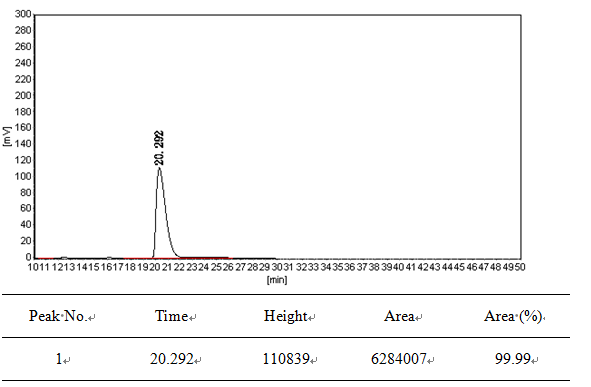


Racemic of **9**


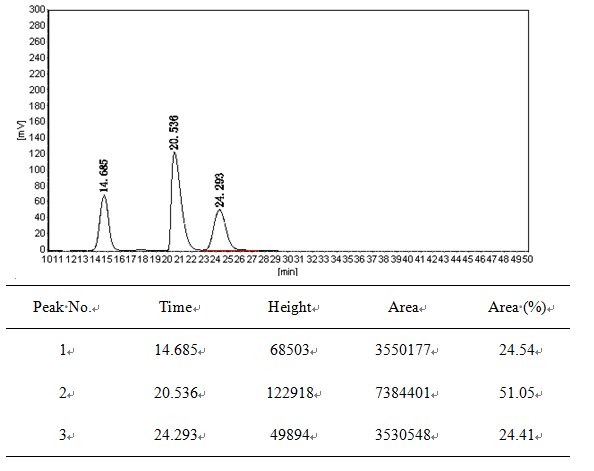


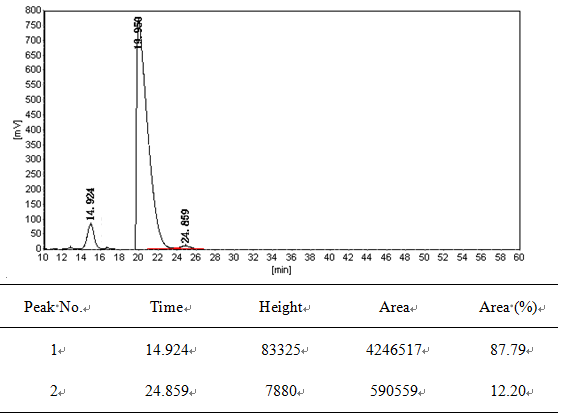


Racemic of **10**


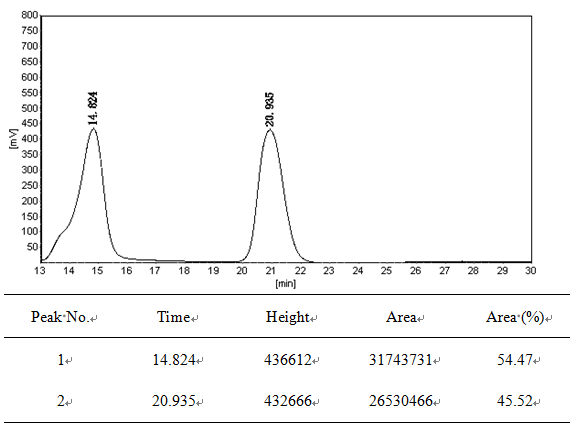


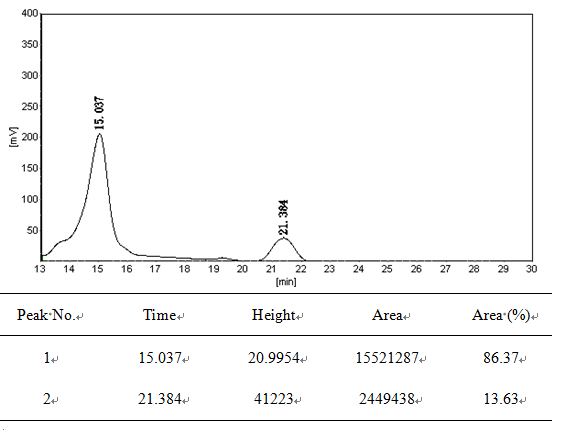


**6. UV-vis spectra of S in presence of Fe^3+^**


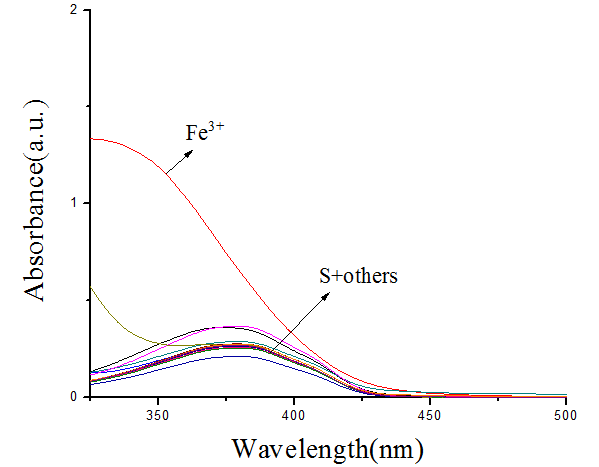


Others metal ions: Hg^2+^, Ag^+^, Ca^2+^, Cu^2+^, Co^2+^ Ni^2+^, Cd^2+^, Pb^2+^, Zn^2+^, Cr^3+^ and Mg^2+^ with their perchlorate salts.

**7. ^1^H NMR titration, Mass spectra and CD spectra**


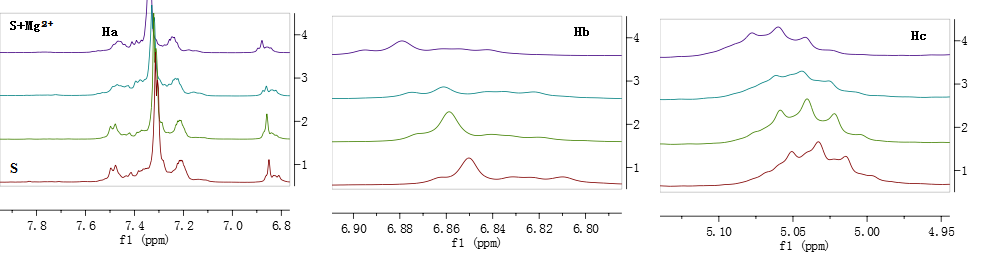


**Figure S2. ^1^H NMR spectra of S with Mg^2+^ ions in DMSO-d_6_.**

[(S)Mg^Ⅱ^+H]^+^

**Figure S3. HRMS spectrum of [(S)Mg^Ⅱ^+H]^+^**

**
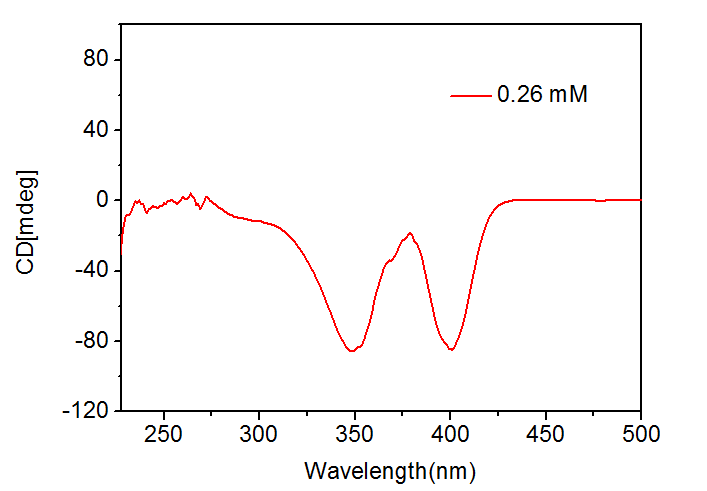
**

**Figure S4. CD spectra of NADH model S**

**8. Data of Molecular modeling via molecular dynamics followed by energy minimization with Gaussian 03**


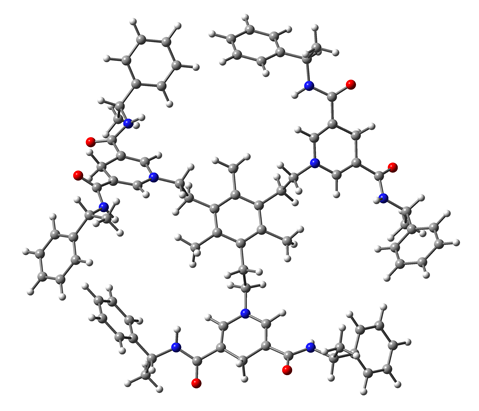


corrdinates (b3lyp/6-31g**) E(RB3LYP) = -4201.1255841600

C -0.278242 1.361868 -0.634719

C -1.119398 0.230032 -0.558094

C -0.560842 -1.043654 -0.316326

C 0.841909 -1.196612 -0.227109

C 1.681941 -0.071926 -0.352267

C 1.118776 1.207798 -0.540786

C 3.194384 -0.212992 -0.317846

H 3.633952 -0.031465 -1.307764

H 3.659335 0.500417 0.369757

H 3.518367 -1.203789 -0.000225

C -0.857161 2.750561 -0.852756

H -0.359042 3.499776 -0.230826

H -0.745896 3.077788 -1.895725

H -1.918316 2.807745 -0.609503

C 2.028231 2.421945 -0.646273

H 1.571084 3.205840 -1.255653

H 2.958787 2.157575 -1.158183

C 2.386226 3.026700 0.733552

H 2.876509 2.270385 1.358833

H 1.474458 3.319342 1.262884

C 2.751010 5.482833 0.845026

C 4.576348 4.066524 0.270890

C 3.465615 6.601550 0.600372

H 1.757175 5.516044 1.279832

C 5.390565 5.115462 0.032121

H 4.919204 3.040955 0.177336

N 3.245740 4.201282 0.644989

C 4.882061 6.543781 0.057697

H 4.938842 6.982434 -0.949462

H 5.533718 7.179417 0.669328

C 6.804108 4.922252 -0.386822

C 2.946868 7.951960 0.951118

O 7.387451 5.772067 -1.058081

O 3.710929 8.897739 1.138088

N 1.576823 8.084893 1.081852

H 1.010999 7.451265 0.533683

N 7.415565 3.739800 -0.011861

H 7.076290 3.312601 0.839673

C 8.847139 3.533186 -0.301563

H 9.364870 4.483730 -0.121371

C 0.976885 9.406596 1.338130

H 1.457905 10.137209 0.674553

C -0.506881 9.349810 1.016336

C -1.079903 10.268032 0.129220

C -1.337352 8.391830 1.618448

C -2.448496 10.238084 -0.147014

H -0.448489 11.014062 -0.347671

C -2.702972 8.353767 1.338339

H -0.909789 7.676986 2.317621

C -3.264362 9.279649 0.455738

H -2.874633 10.963137 -0.835621

H -3.332525 7.606790 1.815137

H -4.329000 9.249994 0.241632

C 9.407227 2.482032 0.641348

C 8.858203 1.191408 0.696286

C 10.496824 2.779381 1.467812

C 9.386272 0.226046 1.554276

H 8.012759 0.941493 0.058966

C 11.031858 1.814199 2.323381

H 10.929845 3.776494 1.440139

C 10.477658 0.534817 2.369505

H 8.953736 -0.770708 1.581609

H 11.879298 2.064694 2.955952

H 10.892026 -0.217864 3.034586

C 9.053558 3.165721 -1.780299

H 10.121472 3.046500 -1.993240

H 8.545389 2.226809 -2.025864

H 8.655721 3.961460 -2.414684

C 1.234781 9.851020 2.788010

H 0.780180 9.150267 3.496481

H 2.310076 9.898035 2.972885

H 0.802452 10.843002 2.958262

C 1.431640 -2.586986 -0.050332

H 2.272175 -2.575191 0.651607

H 0.700495 -3.268424 0.388055

C -1.444079 -2.268300 -0.135896

H -1.254318 -3.031002 -0.901485

H -1.266674 -2.744035 0.835964

H -2.507775 -2.038902 -0.178644

C -2.623033 0.382223 -0.751773

H -3.046910 -0.517436 -1.203214

H -2.828515 1.181542 -1.469437

C -3.381350 0.664745 0.565657

H -3.062798 -0.051090 1.328262

H -3.114431 1.661790 0.944097

C 1.909163 -3.207707 -1.386756

H 1.098682 -3.175061 -2.122486

H 2.734802 -2.621314 -1.804779

C -5.542191 1.389589 -0.405253

C -5.552944 -0.345561 1.224694

C -6.878487 1.313938 -0.576288

H -4.938162 2.114856 -0.940054

C -6.885496 -0.525879 1.110215

H -4.967453 -0.851711 1.985377

N -4.831599 0.545644 0.439965

C -7.710229 0.235150 0.089204

H -8.590495 0.667495 0.579706

H -8.122776 -0.443429 -0.672373

C -7.639120 -1.389028 2.060340

C -7.594838 2.198267 -1.533794

O -8.819961 -1.156688 2.323390

O -8.633393 1.826637 -2.078707

N -7.032183 3.431664 -1.801037

H -6.466003 3.837424 -1.068023

N -6.950354 -2.433058 2.640187

H -6.128799 -2.776588 2.160957

C -7.578198 -3.333261 3.617260

H -8.486869 -2.803763 3.918193

C -7.753945 4.384938 -2.665244

H -8.822212 4.295730 -2.432780

C -7.302666 5.802500 -2.359438

C -8.236312 6.773588 -1.977875

C -5.955485 6.177349 -2.474955

C -7.841110 8.088869 -1.728504

H -9.283092 6.496114 -1.879588

C -5.554533 7.488966 -2.218720

H -5.216038 5.439405 -2.777774

C -6.498649 8.450013 -1.849259

H -8.581920 8.829369 -1.438918

H -4.506866 7.763128 -2.309748

H -6.190268 9.475501 -1.662957

C -8.007007 -4.650215 2.966078

C -7.494110 -5.894319 3.348393

C -8.968269 -4.608599 1.941744

C -7.927637 -7.069600 2.725530

H -6.758463 -5.962601 4.143764

C -9.396521 -5.777146 1.315817

H -9.385902 -3.648439 1.650449

C -8.876593 -7.015842 1.705763

H -7.526946 -8.027420 3.048427

H -10.145956 -5.723937 0.530335

H -9.216922 -7.928963 1.224737

C -6.669516 -3.480021 4.842153

H -7.141742 -4.100625 5.610888

H -5.705537 -3.936059 4.584506

H -6.471119 -2.493265 5.270488

C -7.569061 4.017785 -4.146968

H -6.514728 4.077981 -4.437678

H -7.928099 3.000050 -4.319237

H -8.141405 4.704200 -4.780375

C 1.424322 -5.628634 -1.396475

C 3.573776 -4.891056 -0.689409

C 1.667241 -6.899030 -1.014654

H 0.501182 -5.341778 -1.890127

C 3.941270 -6.133950 -0.313826

H 4.227113 -4.034493 -0.555939

N 2.343181 -4.594208 -1.260695

C 2.995862 -7.316371 -0.413085

H 2.856721 -7.767315 0.578915

H 3.436269 -8.121262 -1.017039

C 5.260998 -6.392340 0.321342

C 0.679701 -7.988626 -1.252671

O 5.425637 -7.347384 1.079037

O 1.032585 -9.165616 -1.309324

N -0.638454 -7.610888 -1.416203

H -0.913762 -6.740532 -0.982671

N 6.277309 -5.496182 0.042661

H 6.227661 -5.031599 -0.854298

C 7.637529 -5.743795 0.555534

H 7.854349 -6.813102 0.436669

C -1.704572 -8.624772 -1.522318

H -1.502521 -9.406046 -0.778080

C -3.034991 -7.966975 -1.193813

C -3.791079 -8.397595 -0.097358

C -3.529729 -6.910788 -1.974633

C -5.012627 -7.795479 0.210730

H -3.418146 -9.212157 0.519474

C -4.748423 -6.305048 -1.666969

H -2.959240 -6.565594 -2.833734

C -5.495523 -6.746174 -0.572109

H -5.588327 -8.139357 1.065275

H -5.118547 -5.491941 -2.286567

H -6.447109 -6.281269 -0.329259

C 8.638256 -4.945270 -0.262161

C 8.524293 -3.551826 -0.384963

C 9.709709 -5.585275 -0.895514

C 9.458040 -2.819553 -1.118711

H 7.698301 -3.037583 0.101637

C 10.649086 -4.855577 -1.626969

H 9.808918 -6.665166 -0.814107

C 10.525716 -3.470604 -1.740954

H 9.359764 -1.739900 -1.196221

H 11.474596 -5.371409 -2.110229

H 11.255586 -2.900841 -2.309534

C 7.719513 -5.416916 2.056105

H 8.725278 -5.631923 2.433075

H 7.501285 -4.358920 2.237820

H 6.999687 -6.028346 2.605116

C -1.693569 -9.288646 -2.908255

H -1.879195 -8.553746 -3.698706

H -0.722197 -9.757433 -3.081658

H -2.471909 -10.057594 -2.961975
